# Ventilator associated pneumonia and infection control

**DOI:** 10.1186/1476-0711-5-7

**Published:** 2006-04-06

**Authors:** Emine Alp, Andreas Voss

**Affiliations:** 1 Radboud University Nijmegen Medical Centre, Nijmegen University Centre for Infections, Nijmegen, The Netherlands; 2 Department of Infectious Diseases, Faculty of Medicine, Erciyes University, Kayseri, Turkey; 3 Canisus Wilhelmina Hospital, Nijmegen, The Netherlands

## Abstract

Ventilator associated pneumonia (VAP) is the leading cause of morbidity and mortality in intensive care units. The incidence of VAP varies from 7% to 70% in different studies and the mortality rates are 20–75% according to the study population. Aspiration of colonized pathogenic microorganisms on the oropharynx and gastrointestinal tract is the main route for the development of VAP. On the other hand, the major risk factor for VAP is intubation and the duration of mechanical ventilation. Diagnosis remains difficult, and studies showed the importance of early initiation of appropriate antibiotic for prognosis. VAP causes extra length of stay in hospital and intensive care units and increases hospital cost. Consequently, infection control policies are more rational and will save money.

## Introduction

Nosocomial pneumonia (NP) is defined as parenchymal lung infection, occurring after the first 48 hours of hospital admission [1]. It accounts for 13–18% of all hospital-acquired infections, but leading cause of death from nosocomial infections [2]. It is a major threat to patients admitted intensive care units (ICU) and receiving mechanical ventilation (MV). In the recent studies, it was shown that ventilator associated pneumonia (VAP) was the most common infectious complication among patients admitted ICU [3,4]. It results in high mortality and morbidity, prolonged lengths of hospitalisation, and also increased cost of hospitalisation. The mortality rates for VAP range from 20% to 75% according to the study population [5-13]. The average excess cost of pneumonia was estimated to be U.S.$1255 per patient in 1982 (14), U.S.$2863 per patient in 1985 [15], and in a recent study in 1999, it was >U.S.$40 000 per patient [16].

Despite the clinical experience and major advances in diagnostic techniques and management, VAP remains a significant problem for intensivists. In this review, epidemiology, diagnosis and, mainly, infection control of VAP were discussed.

## Epidemiology

### Incidence

In different studies, the incidence of VAP was reported different, depending on the definition, the type of hospital or ICU, the population studied, and the type of rate calculated and varies from 7% to 70% [17-23]. In a large database, 1-day point prevalence study, conducted in 1417 European ICUs, pneumonia accounted for 47% of nosocomial infections [4]. In the National Nosocomial Infections Surveillance System (NNIS), NP accounted for 31% of all nosocomial infections in ICU [24] and in another NNIS data in medical ICUs, it was accounted for 27% [25]. The recent studies reported the device-associated incidence rate 13.2–51 per 1000 ventilator days [20,23,26,27]. Generally, the rates of VAP in surgical ICU were higher than in medical ICUs, depending on the differences in the patient population, surgical disorders, the proportion of patients that needed MV and the duration of ventilation. Kollef et al. [28] reported incidences of NP of 21.6% in patients admitted to a cardiothoracic ICU, 14% in other surgical ICU, and 9.3% in a medical ICU.

### Pathophsiology

NP may occur by four routes; haematogenous spread from a distant focus of infection, contiguous spread, inhalation of infectious aerosols and aspiration. Aspiration of the pathogenic gram-positive and gram-negative bacteria, colonized on the oropharynx and gastrointestinal tract, is the main route. The role of other routes is very rare [1].

Once microorganisms reach the distal lung, they multiply and cause invasive disease. The host defence, including filtration and humidification of air in the upper airways, epiglottic and cough reflexes, ciliary transport by respiratory epithelium, phagocytes and opsonins in distal lung, and systemic cell mediated and humoral immunity, prevent bacterial invasion [29]. In ICU, the host defence of patients are usually altered because of their underlying diseases, and devices that are used. They can not cough efficiently due to sedation or underlying disease. And also, when they are intubated, the endotracheal tube holds the vocal cords open and facilitates aspiration.

### Risk factors

The most important risk factor for NP is tracheal intubation; associated with a 3 to 21 fold risk [30-33]. It increases the risk by; 1) causing sinusitis and trauma to nasopharynx (nasotracheal tube), 2) impairing swallowing of secretions, 3) acting as a reservoir for bacterial proliferation, 4) increasing bacterial adherence and colonization of airways, 5) requiring the presence of a foreign body that traumatizes the oropharyngeal epithelium, 6) causing ischemia secondary to cuff pressure, 7) impairing ciliary clearance and cough, 8) causing leakage of secretions around the cuff, and 9) requiring suctioning to remove secretions [34]. Microorganisms can adhere to the surface of the endotracheal tube and some species exude an exopolysaccharide that acts as a slime-like adhesive. That microbial biofilm on the tube surface provides a reservoir of microorganisms, and they are greatly resistant to the action of antimicrobials and host defence [35]. Also, the patient requiring MV exposes to other devices, such as nebulizers, humidifiers, which can be the source of microorganisms.

The duration of MV increases the risk of infection. Cook et al. [22] reported a cumulative increased risk of VAP with time, with 3% per day in the first week of MV, 2% per day in the second week, and 1% per day in the third week. In other studies, similarly, it was shown that the risk of pneumonia increased by the duration of MV and the highest risk was during the first 8–10 days [36-38]. The need for reintubation, urgent intubation and documented massive aspiration are also associated with high incidence of VAP [1,21,29,39].

The effect of prior antibiotic therapy is still controversial. Sirvent et al. [40] reported that a short course of cephalosporin prophylaxis was associated with a lower rate of VAP in patients with structural coma. Also other investigators showed that antibiotics administered during the first days, reduced the risk of early-onset ventilator associated pneumonia [22,37]. However, prior antibiotic exposure predisposes patients to subsequent colonization and infection with resistant pathogens [28,41].

Nasogastric tubes impair the function of the gastroesophageal sphincter and increase the risk of maxillary sinusitis, oropharyngeal colonization and reflux, all of which may lead to migration of bacteria and pneumonia [29,39]. Enteral nutrition given by nasogastric tube is also associated with increased risk of VAP. Moreover, it may predispose to VAP by elevating gastric pH, leading to gastric colonization and increasing the risk of reflux and aspiration by causing gastric distension [22,42,43]. Also patient transportation was found risk factor for VAP by facilitating the aspiration of contaminated secretions from the upper airway and the ventilator circuits in the supine position [44].

The other independent risk factors for VAP are shown in Table 1[6,7,11,22,28,33,34,45]. Identifying these risk factors will guide to prevention measures of VAP.

**Table 1:** Risk factors for ventilator associated pneumonia

**Host factors**	**Intervention factors**	**Other factos**
Age ≥ 60 yr	Duration of MV	Season: fall, winter
Severity of illness	Reintubation	
Organ failure	PEEP	
Poor nutritional state or hypoalbuminemia	Frequent ventilator circuit changes	
Upper abdominal or thoracic surgery	Nasogastric tube	
ARDS	Intracranial pressure monitoring	
Chronic lung disease	Paralytic agents, sedation	
Neuromuscular disease	H_2 _blockers ± antacids	
Trauma, burns	>4 units of blood products	
Coma, depressed level of consciousness	Supine head position	
Large-volume aspiration	Transport out of the ICU	
Upper respiratory tract colonization		
Gastric colonization and pH		
Sinusitis		

### Etiologic agents

The causative organisms vary according to the patients' demographics in the ICU, methods of diagnosis, the durations of hospital and ICU stays, and the antibiotic policy. Gram negative bacteria are the most common pathogens cause VAP in several studies [7,17,44]. In NNIS data, although the most frequent reported isolate was *Staphylococcus aureus *(17%), 59% of all reported isolates were gram-negative. The most common gram-negative species were *Pseudomonas aeruginosa *(15.6%), *Enterobacter *species (10.9%), and *Klebsiella pneumoniae *(7.0%) (24). In recent years, gram-positive bacteria have become more common in ICU and in the EPIC study, *S. aureus *accounted for 31% of the 836 cases with identified microorganisms (46). NNIS data from medical ICU, also, reported high percentage (20%) of *S. aureus *[25]. Polymicrobial infection rate is usually high in VAP [12,17,47,48].

The duration of MV and the prior exposure to antimicrobials significantly influence the distribution patterns of etiologic agents. In early onset VAP (<5 days), methicillin-sensitive *S. aureus*, *Streptococcus pneumoniae*, and *Haemophilus influenzae *are the most common pathogens, whereas methicillin-resistant *S. aureus *(MRSA), *P. aeruginosa, Acinetobacter baumannii *and *Stenotrophomonas maltophilia *are more frequent in late onset VAP (≥ 5 days) [49,50]. Also, MRSA, *P. aeruginosa, A. baumannii *and the other multi-resistant gram negative pathogens are the most common pathogens in the patients expose to prior antibiotic.

The other special factors that predispose patients to infection with specific microorganisms are summarized in Table 2[29,51-56]. Determining risk factors for microorganisms will help to select appropriate antimicrobial treatment, that improves the outcome.

**Table 2:** Risk factors for spesific microorganisms

**Microorganism**	**Risk factor**
*H. influenzae, Moraxella catarhalis, S. pneumoniae*	Chronic obstructive pulmonary disease (COPD), early-onset VAP
*P. aeruginosa, A. baumannii*	Corticosteroid therapy, malnutrition, lung disease (bronchiectasis, cystic fibrosis), late-onset VAP, prior antibiotic exposure
*S. aureus*	Coma, head-trauma, neurosurgery, diabetes mellitus, chronic renal insufficiency, influenza
Anaerobes	Aspiration
*Legionella*	Chemotherapy, corticosteroid therapy, malignancy, renal insufficiency, neutropenia, contamination of (hospital) water system
*Aspergillus*	Corticosteroid therapy, cytotoxic drugs, COPD
*Candida albicans*	Immunosuppression, cytotoxic drugs, corticosteroid therapy, broad-spectrum antibiotics
Influenza	Winter season, immunosuppression, chronic underlying disease
*Respiratory syncytial virus*	Immunosuppression, chronic cardiac or pulmonary disease

## Diagnosis

The diagnosis of pneumonia in mechanically ventilated patients is difficult, and still there is no "gold-standard" diagnostic method. It is usually based on the combination of clinical, radiological, and microbiological criteria defined by Centers for Disease and Control (CDC) (Table 3). But these criteria have low sensitivity and specificity. The systemic signs (fever, leukocytosis, etc.) of infection can be seen by any condition in ICU (pulmonary edema, pulmonary infarction, after surgery, trauma, devascularized tissue, open wounds, etc.). Investigators reported that the clinical diagnosis of VAP is associated 30–35% false-negative and 20–25% false-positive results [57-59]. And also, ICU patients do not always have systemic signs of infection due to their underlying disease (chronic renal failure, immunosuppresion, etc.). Radiological infiltration has limited value, mimicking by cardiogenic pulmonary edema, noncardiogenic pulmonary edema, adult respiratory distress syndrome (ARDS), atelectasis, pulmonary contusion, which are not uncommon in ICU [29]. In an autopsy-proven VAP study, Wunderink et al. [1] reported that no radiographic sign had a diagnostic accuracy greater than 68%. The presence of air bronchograms was the only sign that correlated well with pneumonia, correctly predicting 64% of pneumonias in the entire group. The upper respiratory tract of patients is colonized with potential pulmonary pathogens a few hours after intubation [48,61]. Consequently, isolation of pathogens from tracheal secretions do not always indicate pulmonary infection. But a positive Gram's stain may guide the initial antibiotic therapy. However prior antibiotic and corticosteroid therapy can reduce the sensitivity of this technique [62,63].

**Table 3:** CDC criteria for ventilator associated pneumonia

Three or more of the following criteria:

Rectal temperature >38°C or <35.5°C
Blood leucocytosis (>10.10^3^/mm^3^) and/or left shift or blood leukopenia (<3.10^3^/mm^3^)
More than ten leukocytes in Gram stain of tracheal aspirate (in high power field)
Positive culture from endotracheal aspirate
AND
New, persistent, or progressive radiographical infiltrate

Pugin et al. [64] proposed to combine the seven variables (temperature, leukocytes, tracheal aspirate volume and purulence of tracheal secretions, chest X-ray, oxygenation-PaO_2_/FiO_2_- and semiquantitative culture of tracheal aspirate) for the diagnosis of VAP, defined as clinical pulmonary infection score (CPIS) (Table 4). The score varied from 0 to 12 points and was reported that a CPIS of more than six was associated with a sensitivity of 93% and a specificity of 100% for the diagnosis of pneumonia. In a post mortem study, Papazian and colleagues [65] reported a high diagnostic accuracy of CPIS at a threshold of 6 (72% sensitivity and 85% specificity). However, the original scoring system has some limitations; that it requires 24–48 hours for the results of tracheal aspirate cultures, and also identifying pulmonary infiltrates progression depends on intensivist experience. Singh et al. [66] used a modified CPIS (calculated at baseline from the first five clinical variables, and CPIS at 72 hours was based on all variables of the score) that antibiotics were stopped in patients with a persistent low score (<6) after 3 days of therapy, avoiding unnecessary use of antibiotics, and all patients who discontinued the therapy improved. In a recent study, Fartoukh et al. [67] reported that the modified CPIS does not perform better when the clinical suspicion of pneumonia is high, so they proposed incorporating the results of specimens gram stain (by adding two more points when gram stains were positive) to modified CPIS to increase the sensitivity of the score and the physicians' diagnostic accuracy.

**Table 4:** Clinical pulmonary infection score

Temperature, °C	≥ 36.5 and ≤ 38.4	0 point
	≥ 38.5 and ≤ 38.9	1 point
	≥ 39.0 and ≤ 36.0	2 point
Blood leucocytosis, mm^3^	≥ 4000 and ≤ 11 000	0 point
	<4000 and >11 000	1 point
	+band forms ≥ 500	+ 1 point
Tracheal secretions	<14+ of tracheal secretions	0 point
	≥ 14+secretions	1 point
	+purulent sputum	+1 point
Oxygenation: PaO_2_/FiO_2_, mmHg	>240 or ARDS	0 point
	≤ 240 and no ARDS	2 point
Chest X-ray	No infiltrate	0 point
	Diffused, or patchy infiltrate	1 point
	Localized infiltrate	2 point
Culture of tracheal aspirate (semi-quantitative: 0-1-2 or 3+)	≤ 1 or no growth	0 points
Pathogenic bacteria cultured	>1+	1 point
	>1+ and same pathogenic bacteria seen in Gram stain	2 point

Qualitative cultures of tracheal aspirate (TA) is not a specific diagnostic method because of the lower respiratory tract colonization and a high percentage of false-positive results [48,68]. However, investigators reported that quantitative cultures of TA have equal diagnostic accuracy to the other invasive techniques [69-73]. In a recent study, quantitative cultures of TA were compared with plugged telescoping catheter (PTC). The specificity of TA was similar to PTC when a cut-off point of 10^6 ^cfu/mL or higher was used, although the sensitivity of TA at ≥ 10^6 ^cfu/mL was lower than PTC. But when a cut-off point of 10^5 ^cfu/mL was used, the sensitivity of TA was not statistically different from that of PTC [74]. Although, quantitative cultures of TA is non-invasive, inexpensive and a simple method, it has some risks, that if the cut-off value ≥ 10^6 ^cfu/mL is used, sensitivity will be low and some patients with VAP may not be identified or when the cut-off value ≥ 10^5 ^cfu/mL is used, unnecessary antibiotic treatment will be given because of low specificity [75].

In the recent years, many investigators favour invasive techniques for diagnosis of pneumonia (protected-specimen brush -PSB- or bronchoalveolar lavage-BAL-) that may have more diagnostic accuracy [76-80]. In PSB, 0.001 mL of secretions are collected and the presence of >10^3 ^cfu/mL bacteria has 80–90% sensitivity and 95% specificity for the diagnosis of VAP. In BAL, larger proportion of lung can be sampled and the diagnostic threshold is >10^4 ^cfu/mL. The sensitivity and specificity of BAL are 86–100% and 95–100%, respectively [76,81-83]. Heyland and colleagues [84] proposed that PSB or BAL may increase physician confidence in the diagnosis and management of VAP and allows for greater ability to limit or discontinue antibiotic therapy. Also in this study, patients who underwent bronchoscopy with PSB and BAL had a lower mortality rate compared with patients who did not undergo bronchoscopy. However, a recent meta-analysis concluded that regular use of bronchoscopy for the diagnosis of VAP does not alter mortality, because it does not directly affect the initial antibiotic prescription [85]. The disadvantages of these invasive techniques are [71,86,87]; a) prior antibiotic use may decrease the sensitivity and accuracy of these methods. However, in a recent study, Souweine et al. [88] reported that if a current antibiotic treatment prescribed for a prior infectious disease other than VAP, the diagnostic accuracy of protected specimen brush or bronchoalveolar lavage is not changed, b) these techniques are based on quantitative culture and results of these cultures require 24–48 hours, and, therefore miss early cases, and also give no information about appropriate initial antibiotic therapy, c) these invasive tests may worsen the patient's status (cardiac arrhythmias, hypoxemia, bleeding, etc.), d) increase the costs of caring, e) it has not been proven that the use of these invasive techniques lead to a decrease in patients' mortality.

The spread of microorganism to blood or pleural space is <10%, so blood and pleural effusion cultures have low sensitivity and specificity. Luna and colleagues [89] demonstrated that the positive predictive value of blood cultures to detect the etiologic microorganism was 73% and the sensitivity of blood cultures was only 26%. They concluded that blood cultures in patients with VAP are useful if there is suspicion of another probable infectious condition, but the isolation of a microorganism in the blood does not confirm that microorganism as the pathogen causing VAP. Therefore, two sets of blood samples for culture and tapping pleural effusions >10 mm should be performed in patients suspected VAP [50].

In conclusion, microbiological testing should be always performed to decide the appropriate initial empirical antibiotic therapy. Clinicians can choose optimal diagnostic test for specific patients in their clinical setting.

## Infection control

Because of longer duration of mechanical ventilation, longer stay in the ICU, increased use of antibiotics, higher costs for healthcare, and most importantly, increased mortality, the prevention of VAP is the major priority. But, despite the advances in the pathogenesis of VAP, intensivists still struggle with the prevention strategy.

### Hand hygiene

Basic hygiene principles of infection control (hand washing/disinfection just before and after each patient contact, the use of glove and sterile equipment) remain important for the prevention of VAP. Healthcare workers (HCW) can spread microorganisms from patient to patient by their hands easily. Although HCWs realize the importance of handwashing/disinfection, their compliance is still low (25–40%) [90-92]. Especially their compliance rate is lowest in activities that carried higher risk for transmission and in ICU. High workload decreases their compliance [93]. Wrist watches, bangles, and other jewellery act as reservoirs for organisms, and inhibit effective hand cleaning [94]. Therefore, staff have to take off wrist watch and jewellery to achieve effective hand cleaning. They have to use gowns and gloves when appropriate and must change and wash/disinfect their hands between patients [95]. Bedside hand antiseptics (alcohol-based handrub solution), easier access to sinks and availability of washing equipment, decrease in workload, communication and education tools (posters) and feedback improve compliance and decrease the cross-transmission of nosocomial infection [90,96].

### Ventilator equipment

The internal machinery of mechanical ventilators is not an important risk factor for VAP. Therefore, using a filter between the inspiratory phase circuit and the patient is not necessary. Furthermore, the importance of filters on the expiratory limb of the mechanical-ventilator circuit in preventing cross comtamination has not known and needs further investigation [34].

The devices used on the respiratory tract come into contact with mucous membranes, therefore cleaning and high-level disinfection (at 75°C for 30 minutes) of reusable equipments are required [97]. Resuscitation bags, spirometers, and oxygen analyzers must be cleaned and disinfected between patients to avoid cross-transmission [95].

In several studies, routine changing of the ventilator circuits is not recommended [26,98-104]. Replacement is required when there is gross soilage and mechanical malfunction [105]. The condensate fluid in the ventilator circuit, which contains high concentration of pathogenic bacteria and a risk factor for VAP, has to be removed regularly [106]. And accidental drainage of condensate into the patient's airway and contamination of caregivers during ventilator disconnection or during disposal of condensate should be avoided. In-line devices with one-way valves, put in place into disposable circuits and emptied regularly, are recommended for collecting condensate [75].

Humidification of the inspired air is an important care in the ventilator management. Humidification may be achieved by active humidifiers (bubble-through or wick) or passive humidifiers (hygroscopic condenser-artificial nose- or heat-moisture exchanger-HME-). In humidification, formation of condensate in tubing and colonization of this condensate with microorganisms is an important risk factor for VAP. HME recycle heat and moisture that reduces the condensate formation and also bacterial colonization in the circuit. Moreover they have bacterial filtration characteristics [34,98]. They do not need to be changed daily and can be used for at least 48 hours, sometimes for up to 1 week [107,108]. Also with other advantages (reduced nurses workload, reduced financial cost, and better safety), HMEs are favourable devices in many ICUs. Indeed several investigators [109-112] reported lower rates of VAP in HME groups than conventional heated-water humidification systems, the effect of HME on the prevention of VAP is still controversial and a recent study showed no significant difference in VAP rates [113]. Furthermore, HMEs increase dead space and resistance to breathing, cause airway occlusion, and more tenacious secretions [34,98,104,111]. Additional studies are needed to identify the benefits of HMEs on infection control of VAP.

Nebulizers are used for medication or humidification of air, and inserted into the inspiratory phase of the mechanical ventilator circuit. They can be contaminated by condensate in the tube or by using contaminated solutions, and inoculate infectious aerosol particles directly into the lung parenchyma and can cause outbreaks in ICUs. Recommendations for the infection control of nebulizers are; a) filling immediately before use, b) using sterile water and drugs, c) never refilling the liquid to be nebulised, d) cleaning and disinfecting the receptacle daily, d) using sterile water for rinsing and allowing to dry, e) using patient-specific large-volume nebulizers, f) using patient specific mask, mouthpiece, connecting pieces and medicine cups [34,114].

Suctioning the secretions in the trachea is another approach to VAP prevention. Two types of tracheal suction catheters are used on ventilated patients; the open, single-use catheters and the closed, multiple-use catheters. In single-use system, HCWs have to use sterile solutions during rinsing these catheters and have to care aseptic technique when suctioning endotracheal secretions. In closed suctioning systems, secretions can be suctioned without removal of mechanical ventilation support. This may cause less hypoxia, hypotension and arrhythmias, and also less environmental contamination [115,116]. However similar VAP rates with closed and open system were suggested in the earlier trials [115,117], Combes and colleagues [116] reported a 3.5 times greater risk of VAP in open suctioning system than closed suctioning system in a recent study. Indeed, closed suction catheter is an extension of the ventilator circuit, daily change of this catheter is not necessary for infection control, and in one study no significant difference in VAP rate was reported when daily changes were compared with no routine changes, that may decrease the costs [118]. The use of closed suction system is recommended as part of a VAP prevention program [104].

### Non-invasive ventilation

The relationship between the use of invasive devices and nosocomial pneumonia directed the investigators to use noninvasive ventilation to reduce the VAP rates. In several studies, lower risk of VAP, with less antibiotic use, with a shorter length of ICU stay, and with lower mortality were reported in the use of non-invasive ventilation [119-123]. Therefore, care can be taken to use non-invasive mechanical ventilation more often, and to reduce the frequency of tracheal intubation.

### Endotracheal tubes

Endotracheal tube alters host defences, impairs mechanical clearance from the respiratory tract, causes local trauma and inflammation, and allows pooling of secretions around the cuff. The pressure of the endotracheal tube-cuff should be sufficient to prevent the leakage of colonized subglottic secretions into the lower airway [37]. Also, continuous or intermittent suctioning of oropharyngeal and upper respiratory tract secretions above endotracheal cuffs can prevent aspiration. Endotracheal tubes with a separate dorsal lumen above the cuff, designed to suction the subglottic secretions continuously, were found able to decrease the rates of early-onset VAP [124,125]. However, in another randomised trial, no benefit with continous subglottic suction on the overall VAP frequency was found [126]. It can reduce but not eliminate the volume of fluid aspirated into the lungs. The lack of effect on prevention of late-onset pneumonia and the high cost of these tubes restrict the usage of them.

Microbial biofilm on the endotracheal tube surface is a reservoir for the pathogens and prevent the microorganisms from the action of antibiotics [35]. Adair and colleagues [127] proposed that high concentrations of antibiotic on the endotracheal luminal surface, achieved either by nebuliser or endotracheal surface modification, would be expected to prevent biofilm formation on the endotracheal tube and may have a role in reducing the incidence of VAP, also minimising patient exposure to systemic antibiotics.

Nasotracheal intubation increases the risk of nosocomial sinusitis, that may predispose VAP by the aspiration of infected secretions from nasal sinuses [128,129]. Therefore, endotracheal intubation should be preferred to decrease the risk of VAP.

### Nasotracheal tube and enteral nutrition

As nasotracheal intubation, nasogastric tube can cause orophanryngeal colonization and nosocomial sinusitis. By impairing the function of the upper oesophagus sphincter, it may facilitate the reflux of bacteria from the gut. As a result, it increases the risk of VAP [130,131]. In a randomized study, gastroesophageal reflux and microaspiration of gastric contents to the lower airways were not influenced by the size of the nasogastric tube. Because of their potential complications (tracheal malposition by coiling and clogging) and high cost, the small-bore nasogastric tubes are not routinely recommended for the prevention of VAP [132].

Poor nutritional state and hypoalbuminemia contribute the development of VAP. For this reason, early initiation of enteral nutrition may have preventive effect in mechanically ventilated patients. Moreover, it helps to maintain the gastrointestinal epithelium and reduces the need for stress-bleeding prophylaxis. However, because of using nasogastric tubes and alkalinization of the stomach contents by these feeds; gastric colonization, gastrooesophageal reflux, aspiration and pneumonia might be promoted [29]. In a recent study, postpyloric enteral access placement improved tube-feeding tolerance and reduced the rates of VAP [133]. Heyland et al. [134] used acidified feeds in critically ill patients and demonstrated a dramatic reduction in bacterial growth from aspirates of stomach contents and a lower rate of gram-negative bacterial growth in tracheal secretions in patients receiving acidified feeds, but not a significant reduction in nosocomial pneumonia. They cannot be used in patients with active gastro-intestinal bleeding, acidemia, or renal failure. Furthermore, it was a small size study and further research on the effect of prevention is needed, before it is used in practice.

### Selective digestive decontamination and oral care

In the recent years, selective decontamination of the digestive tract (SDD) is one of the most extensively studied prevention strategy in VAP. In SDD, topical non-absorbed antimicrobials (usually combining polymyxin, aminoglycoside and amphotericin B) are used to prevent gastrointestinal colonisation by pathogenic microorganisms. It selectively eradicates the potential pathogenic microorganisms (gram-negative aerobic intestinal bacteria, *S. aureus *and fungi) and does not affect anaerobic flora, as elimination of anaerobic flora leads to increased colonization with gram-negative aerobic flora. Although some investigators used only topical antibiotics applied to the oropharynx and through a nasogastric tube, many of them added systemic therapy with a broad spectrum (e.g. cefotaxime) during the first few days, to prevent early infections with *S. pneumoniae, H. influenzae *and *S. aureus *[29]. In a recent meta-analysis that searched 33 randomized controlled trials published from 1984 to 1996, significant reductions in the incidence of respiratory tract infections (65%) and in total mortality (20%) were determined [135]. Also in this meta-analysis and in the other recent prospective, randomized studies, it was mentioned that using only topical antibiotics reduced respiratory infections, but did not influence the survival [135-137]. The threat of SDD is to lead the selection and overgrowth of antibiotic resistant microorganisms. In a recent study from the Netherlands, where the incidence of MRSA and vancomycin resistant enterococcus (VRE) are very low, a reduction in the frequency of colonization with resistant gram-negative bacteria and no effect on the acquisition of MRSA were reported (138). But the studies from the ICUs where MRSA was endemic, increased incidence of MRSA was reported (139,140). Therefore, in ICUs with high incidence of multi-resistant microorganisms, SDD cannot be used. On the other hand, in trauma and surgical patients, SDD seems more effective than in medical patients, may be due to less colonisation [141]. In conclusion the routine use of SDD in ICUs is not recommended, it should be decided according to the patient population studied and the characteristic of ICU.

In addition, the colonization of the oral cavity with pathogens is important risk factor for the develoment of VAP, it is unclear if oral care with chlorhexidine reduces VAP. Also the concern over a chlorhexidine-related increase in colonization of gram negative bacteria should be considered [142].

### Body position and drugs

Semirecumbent position (45°) prevents aspiration and the passage of bacteria into the airways, and should be preferred in ICU patients, if there is no contraindication [143].

"Kinetic Beds" or Continous Lateral Rotational Therapy (CLRT) turn continuously and slowly and change the patient's position. Investigators believe that it helps the drainage of pulmonary secretions. However, these beds are so expensive and their effectiveness are not demonstrated. So, the routine use of these beds is not recommended [97]. Also, chest physiotherapy, to improve the clearance of secretions, for the prevention of VAP is not recommended because of its lack of proven benefits and the associated risks (e.g., arterial oxygen desaturation) [105,144].

Stress ulcer prophylaxis is proposed to be a risk factor due to the alkalinization of gastric content. The effect of stress ulcer prophylaxis with H_2_-antagonists or antacids on VAP is still controversial. In some studies [145,146], the use of sucralfate was associated with a decreased incidence of VAP, however the other reports did not support this [147,148]. Also, H_2_-antagonists are more efficient for anti-ulcer prophylaxis than the sucralfate [148]. Therefore, the choice of agent for prophylaxis should be done according to the patient and cost-effectiveness.

To reduce the aspiration of oropharyngeal contents, over use of sedatives should be avoided. Kress et al. [149] reported that for reducing over use of sedatives, daily interruption of sedative-drug infusions until the patients were awake decreased the duration of mechanical ventilation and the length of stay in the ICU.
